# Proteases Inhibition Assessment on PC12 and NGF Treated Cells after Oxygen and Glucose Deprivation Reveals a Distinct Role for Aspartyl Proteases

**DOI:** 10.1371/journal.pone.0025950

**Published:** 2011-10-18

**Authors:** Aristidis Kritis, Chryssa Pourzitaki, Ioannis Klagas, Michael Chourdakis, Maria Albani

**Affiliations:** 1 Laboratory of Physiology, School of Medicine, Aristotle University of Thessaloniki, Thessaloniki, Greece; 2 Laboratory of Pharmacology, School of Medicine, Aristotle University of Thessaloniki, Thessaloniki, Greece; Universidade Federal do Rio de Janeiro, Brazil

## Abstract

Hypoxia is a severe stressful condition and induces cell death leading to neuronal loss both to the developing and adult nervous system. Central theme to cellular death is the activation of different classes of proteases such as caspases calpains and cathepsins. In the present study we investigated the involvement of these proteases, in the hypoxia-induced PC12 cell death. Rat PC12 is a model cell line for experimentation relevant to the nervous system and several protocols have been developed for either lethal hypoxia (oxygen and glucose deprivation OGD) or ischemic preconditioning (IPS). Nerve Growth Factor (NGF) treated PC12 differentiate to a sympathetic phenotype, expressing neurites and excitability. Lethal hypoxia was established by exposing undifferentiated and NGF-treated PC12 cells to a mixture of N_2_/CO_2_ (93:5%) in DMEM depleted of glucose and sodium pyruvate for 16 h. The involvement of caspases, calpains and lysosomal cathepsins D and E to the cell death induced by lethal OGD was investigated employing protease specific inhibitors such as z-VAD-fmk for the caspases, MDL28170 for the calpains and pepstatin A for the cathepsins D and E. Our findings show that pepstatin A provides statistically significant protection from cell death of both naive and NGF treated PC12 cells exposed to lethal OGD. We propose that apart from the established processes of apoptosis and necrosis that are integral components of lethal OGD, the activation of cathepsins D and E launches additional cell death pathways in which these proteases are key partners.

## Introduction

Reduced oxygen supply to the brain causes severe adverse effects such as neurological handicaps due to neuronal cell death from energy shortage, free radicals damage, glutamate induced excitotoxicity, and launches numerous diverse cell survival and death mechanisms. Several in-vitro and in-vivo models have been developed to study conditions that approximate ischemia to the brain. A widely accepted model of ischemia has been developed using PC12 cells exposed in conditions of oxygen and glucose deprivation (OGD). PC12 cells originating from rat pheochromocytoma upon Nerve Growth Factor (NGF) treatment, switch their phenotype from a proliferating, undifferentiated cell to a post-mitotic, differentiated, neurite-bearing NGF-dependent neuron [1]. OGD exposed PC12 cells reportedly suffer from mitochondrial dysfunction, oxidative stress and loss of energy that eventually lead to cell death [2]–[4]. This cell death in some cases is characterised as apoptotic [5], [6] in others as necrotic [7] and in some recent reports, the process of autophagy is implicated in the cell death processes [8], [9]. This situation is also reflected in hypoxic rat brain studies stating that “early neurodegeneration after hypoxia-ischemia in neonatal rat is necrosis while delayed neuronal death is apoptosis” [10]. In some cases a “continuum phenotype” of cell death is reported [11] when apoptosis is hindered from completion possibly due to energy shortage. There are early reports indicating that in ischemia, the activation of the family of cysteine proteases, caspases, is upregulated [12].

On the other hand the abnormal increase of the intracellular Ca^2+^ levels triggers the neuronal damage associated with hypoxia or ischemia. In the vulnerable neurons the endogenous Ca^2+^ levels can be abnormally increased via several routes, resulting in the activation of a number of calcium-sensitive enzymes such as the family of calpain proteases [13]–[15].

Several lines of evidence suggest that calcium-activated proteolysis plays a pivotal role in hypoxic-ischemic neuronal damage. The levels of spectrin, microtubule-associated protein and neurofilament protein, are substantially reduced during and after dense hypoxia or ischemia. These proteins are preferred substrates for the calcium-activated proteases, calpains [16], [17]. Indeed, calpain-mediated proteolysis appears to be one of the earliest biochemical changes occurring after a dense ischemic challenge [18].

While caspases are the key partners for eliciting apoptotic cell death [19], [20], calpains are considered to participate in necrotic cell death [21], [22]. Furthermore a third class of proteases, the lysosomal cathepsins, has a dual role in regulating apoptosis [23]–[25], and executing necrosis [26]. Moreover, the lysosomal cathepsins are constituent members of the autophagic pathways [26], [27].

In the present study we have investigated in detail the roles played by the calpains, caspases and the lysosomal cathepsins, in our PC12-OGD model for lethal hypoxia, by specific inhibition of their activities.

## Results

### Lethal Hypoxia

**Figure 1:**
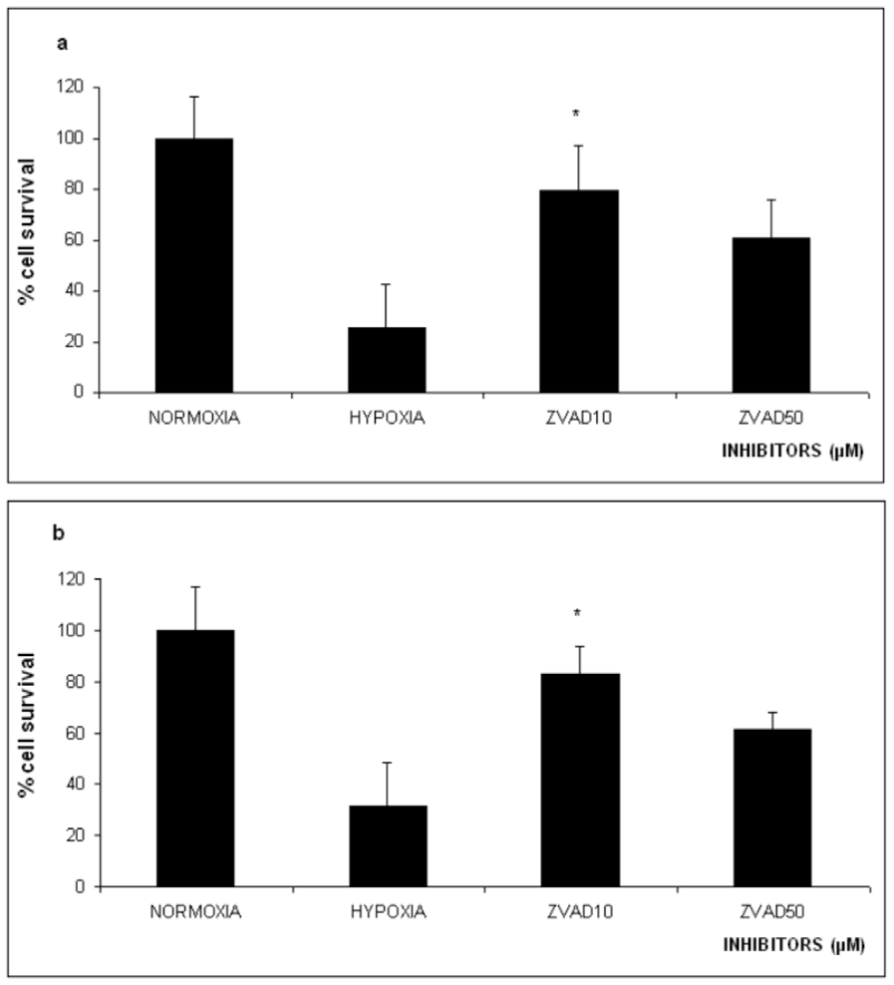
Effect of 16 hours Oxygen-Glucose Deprivation (OGD) on % survival of PC12 cells in the absence or presence of 10 µM and 50 µM of the specific caspase inhibitor z-VAD-fmk. The values are presented as mean ± SEM, *p<0.05, **p<0.01. **Panel a**, naive PC12 cells, **panel b** PC12 cells NGF treated.

PC12 cells were exposed in lethal hypoxia conditions (OGD for 16 hours) as described by [8], [28]. The number of cells survived OGD was initially determined by trypan blue exclusion assay and cell counting on a Neubauer plate (data not shown). After having established that death was due to hypoxia, cell viability was determined by assessment of the integrity of mitochondrial function using the MTT assay. PC12 cells exposed to OGD show a statistically significant reduction in the number of living cells, down to 25.1%±18.3%, p<0.001, compared to PC12 cells growing in complete medium under normoxia (control population 100%), as determined by the MTT assay **Figures 1a** **,** **2a** **,** **3a** **,** **4a** **,** **5a**. Similarly NGF treated PC12 cells exposed to OGD showed a statistically significant reduction in the number of living cells down to 31.6%±22.8, (p<0.001significance) compared to NGF differentiated PC12 cells growing in complete medium under normoxia (control population 100%). **Figure 1b** **,** **2b** **,** **3b** **,** **4b** **,** **5b**.

**Figure 2:**
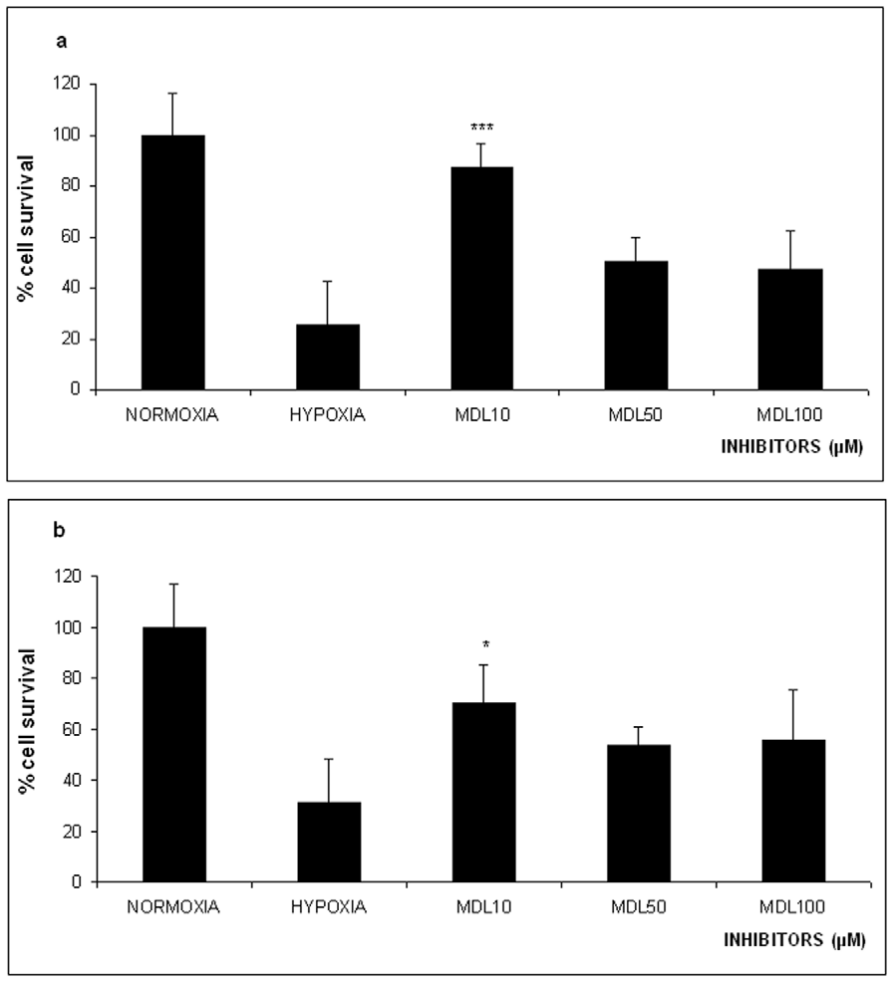
Effect of 16 hours Oxygen-Glucose Deprivation (OGD) on % survival of PC12 cells in the absence or presence of the permeable calpain inhibitor MDL28170 (10 µM, 50 µM and 100 µM). The values are presented as mean ± SEM, *p<0.05, **p<0.01, ***p<0,001. **Panel a**, naive PC12 cells, **panel b** PC12 cells NGF treated.

**Figure 3:**
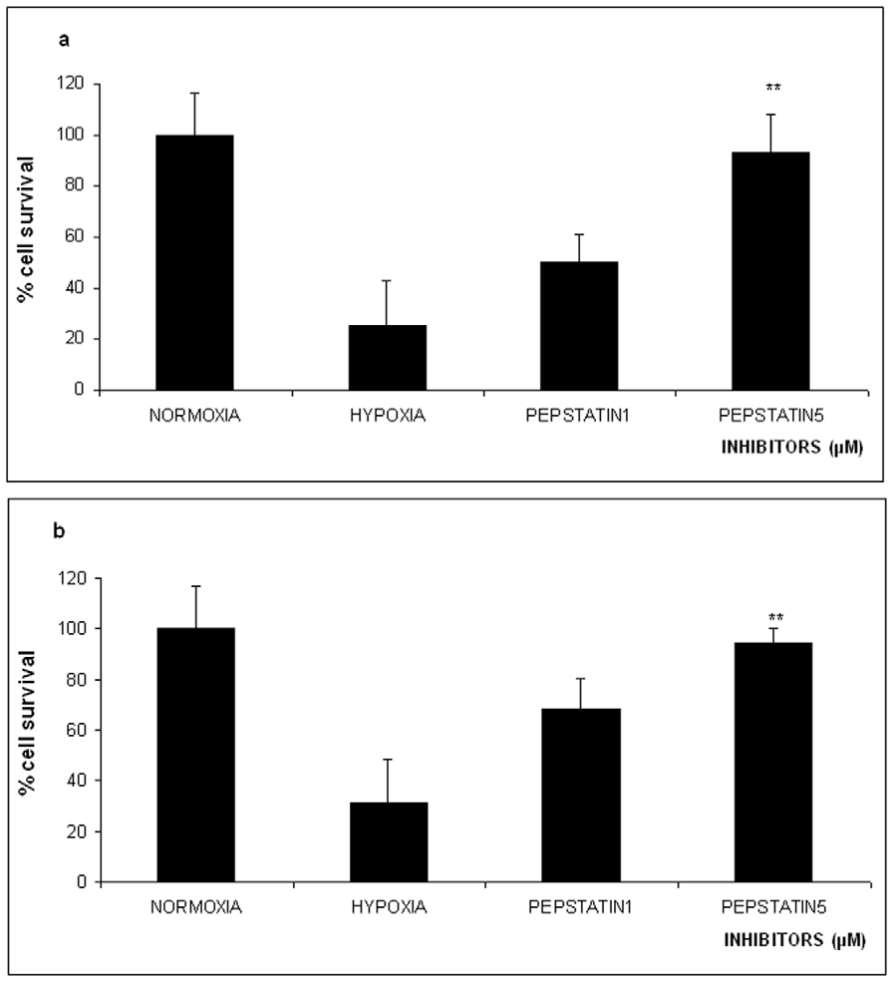
Effect of 16 hours Oxygen-Glucose Deprivation (OGD) on % survival of PC12 cells in the absence or presence of different concentrations of the aspartyl protease inhibitor pepstatin A (1 µM and 5 µM). The values are presented as mean ± SEM, *p<0.05, **p<0.01. **Panel a**, naive PC12 cells, **panel b** PC12 cells NGF treated.

**Figure 4:**
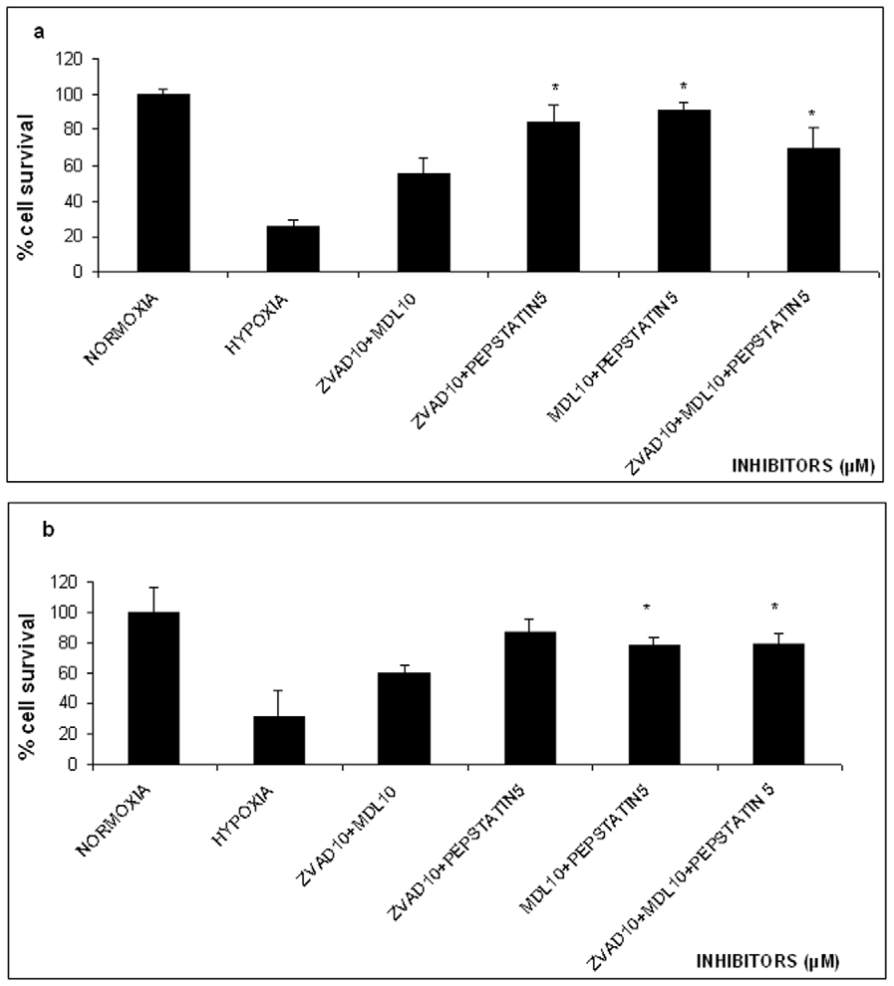
Effect of 16 hours Oxygen-Glucose Deprivation (OGD) on % survival of PC12 cells in the absence or presence of different combinations of caspase, calpain and aspartyl protease inhibition (10 µM/10 µM/5 µM). The values are presented as mean ± SEM, *p<0.05, **p<0.01. **Panel a**, naive PC12 cells, **panel b** PC12 cells NGF treated.

**Figure 5:**
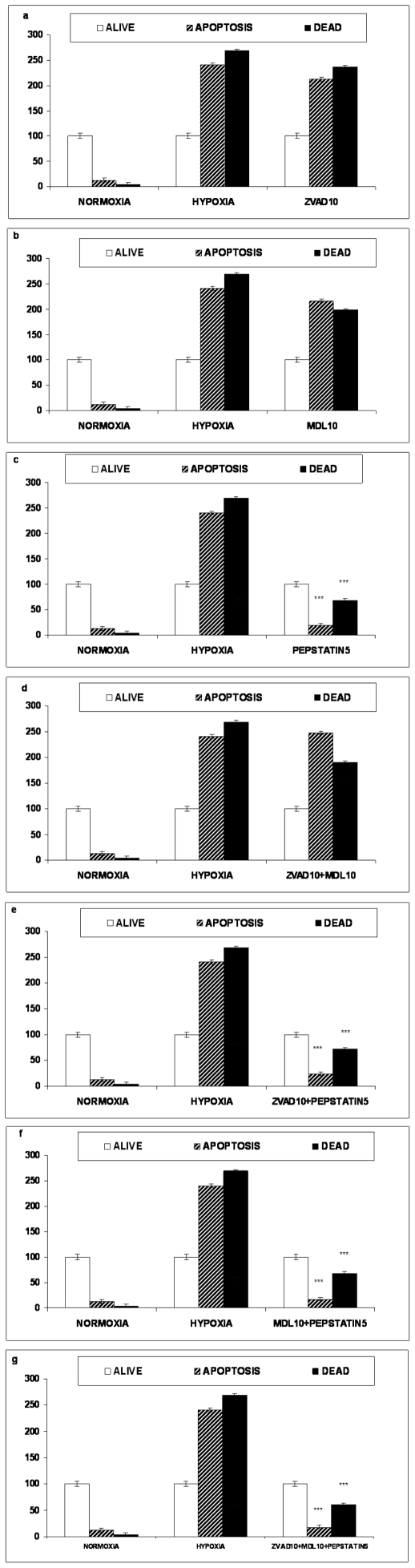
Effect of 16 hours Oxygen-Glucose Deprivation (OGD) on naive PC12 cells tested with Annexin V–FITC/PI cell membrane permeability assay after caspase (10 µM), calpain (10 µM), aspartyl protease (5 µM) inhibition, and their combinations panels a to g. The values are presented as mean ± SEM, *p<0.05, **p<0.01, ***p<0.001.

### Caspases' inhibition

To assess the involvement of the caspases in the cell death caused by lethal hypoxia we exposed naïve and NGF treated PC12 cells to OGD conditions after a 30 min preincubation with different concentrations of the specific caspase inhibitor z-VAD-fmk. Remarkably, the z-VAD-fmk caspase inhibition in a concentration of 10 µM, significantly protects untreated PC_12_ cells from lethal hypoxia (60.7%±17.4 cell survival compared to PC_12_ cells growing in normoxia used as control (p<0.05). **Figure 1a**.

On the other hand lethal hypoxia reduced the cell population of NGF treated cells down to 31.6%±22.8 at p<0.001 significance (compared to the control cells). Perincubation with 10 µM z-VAD-fmk increased the survival of the cells to 83.1%±21.7, p<0.05 significance. In contrast, the use of a higher concentration of the z-VAD-fmk inhibitor (50 µM) was not effective in increasing cell viability in this assay **Figure 1b**.

### Calpains' Inhibition

Next we investigated the calpains involvement in the cell death elicited by lethal OGD. To this end, we have treated naïve and NGF differentiated PC12 cells with different concentrations of the specific calpain inhibitor MDL28170 for 30 min before subjecting them to OGD. The pre-treatment of undifferentiated PC12 cells with 10 µM of MDL18170 results in increased cell viability (86.75%±23.95, p<0.05 significance) and provided statistical significant protection of the treated PC12 cell population against OGD, compared to the control cells. In contrast, cell pre-treatment with higher amounts of MDL18170 (50 µM and 100 µM) failed to provide significant statistical protection of the treated cells against OGD (**Figure 2a**).

Similarly, 10 µM but not 50 µM or 100 µM of the MDL18170 inhibitor, protected from OGD induced cell death the NGF differentiated PC12 cells (70.4%±22.3, p<0.05 significance) compared to untreated control cells **Figure 2b**.

### Cathepsins' Inhibition

Pepstatin A which is an aspartyl protease inhibitor, specific for cathepsins D and E, was used to assess the role of these proteases in OGD induced cell death. Preincubation of both naïve and NGF treated PC12 cells with 5 µM of pepstatin A increased the viability of the naïve and the NGF treated cell population to 93.16%±25.2 and 94.4%±19.8 both at a statistical significance of p<0.01 respectively compared to the control cells (**Figure 4a** **and** **Figure 4b**) and protected both cell populations from lethal OGD.

### Combination of protease inhibitors

We next tested whether the combined use of different protease inhibitors results in increased survival of OGD subjected naïve PC12 cells. A statistically significant survival increase of the lethal OGD exposed PC12 cell was observed in the situation were a) 10 µM z-VAD-fmk in addition to 10 µM MDL28170 were used; we observed 54.8±15% survival at p<0.05 significance of the treated cells compared to control cell population, b) 10 µM z-VAD-fmk in addition to 5 µM Pepstatin A were used; we observed a 84.1±16.3% survival, at p<0.05 significance and c) 10 µM MDL28170 together with 5 µM Pepstatin A were used; we observed a 91.25±14.3% survival at p<0.05 significance (**Figure 4a**).

Additionally, similar results were obtained with NGF differentiated PC12 cell populations preincubated with combinations of the protease inhibitors. Specifically, a) the combined addition of 10 µM z-VAD-fmk and 10 µM MDL28170 resulted in 60.6±17.4% cell viability (at p<0.05 significance), b) the combined addition of 10 µM z-VAD-fmk and 5 µM Pepstatin A resulted in 87.8%±21.3% survival (at p<0.05 significance) and c) 10 µM MDL28170 in addition to 5 µM Pepstatin A raised the living cell number to 77.5±19.1% (at p<0.05 significance) **Figure 4b**.

Remarkably, the combination of all three inhibitors, at the effective concentrations, was marginally effective in improving the survival of either naïve or NGF treated PC12 cells exposed to lethal OGD; we observed a 69.7%±17.2 survival at p = 0.051 for naïve cells (**Figure 4a**) and 78.8±21.3% cell survival for (at p<0.05 significance) for NGF treated PC12 (**Figure 4b**). In either case, the post hoc analysis did not reveal any statistical differences when comparing with the combinations of two inhibitors.

### Flow Cytometry

#### Naive PC12 Cells

Analysis of the results obtained from Annexin V–FITC/PI cell membrane integrity and permeability assay in PC12 cells revealed that under normoxia 85.9±5% of the cell population was alive, 10.4±4% was found to be in an apoptotic stage and 3.6±3% was already dead. These numbers were expressed as percentage of the living cells; 12.2% were in an apoptotic state and 4.2% were dead. After 16 hours of OGD these percentages were 240.2% apoptotic and 268.6% dead (expressed as percent of the living cells in each state i.e. apoptotic or dead). **Figure 5** PC12 preincubated with either 10 µM z-VAD-fmk or 10 µM MDL28170 alone or in combination, show a statically insignificant decrease of both the apoptotic and the dead cell populations percentages **Figures 5a, b, c**.

In contrast, the preincubation with 5 µM Pepstatin A, **Figure 5d**, decreased the apoptotic and dead cell populations of naive PC12 cell exposed to lethal hypoxia measured by this assay to 18.7% and 68.2% respectively in a statistically significant manner, compared to the OGD subjected control cell population (p<0.001).

Preincubation of PC12 cells with 5 µM Pepstatin A in double combinations with the other inhibitors as well as with the combination containing all three inhibitors annul the OGD effect in the alive and apoptotic cell populations in a statistically significant manner. More specifically, the numbers representing the percent of the living cells in each state (apoptotic or dead) were 23.4% apoptotic and 71.8% dead when used in double combination with z-VAD-fmk 10 µM (p<0.001), or 16.7% apoptotic and 68.5% dead when used in double combination with MDL28170 10 µM (p<0.001) or 17.9% apoptotic and 60.7% dead when the triple combination with all inhibitors (p<0.001) expressed as percent of the living cells in each state (apoptotic or dead) ) **Figures 5e, f, g**.

### NGF treated cells

Annexin V–FITC/PI staining coupled with flow cytometry assay of NGF treated cells showed that under normal conditions 87.2% of the total cell population were alive, 9.2% were apparently early apoptotic as they were Annexin V positive and 3.6% were dead cells (PI positive). The same result expressed as the percentage of the living cells is 10.6% of the living cell population were early apoptotic and 4.1% dead.

**Figure 6:**
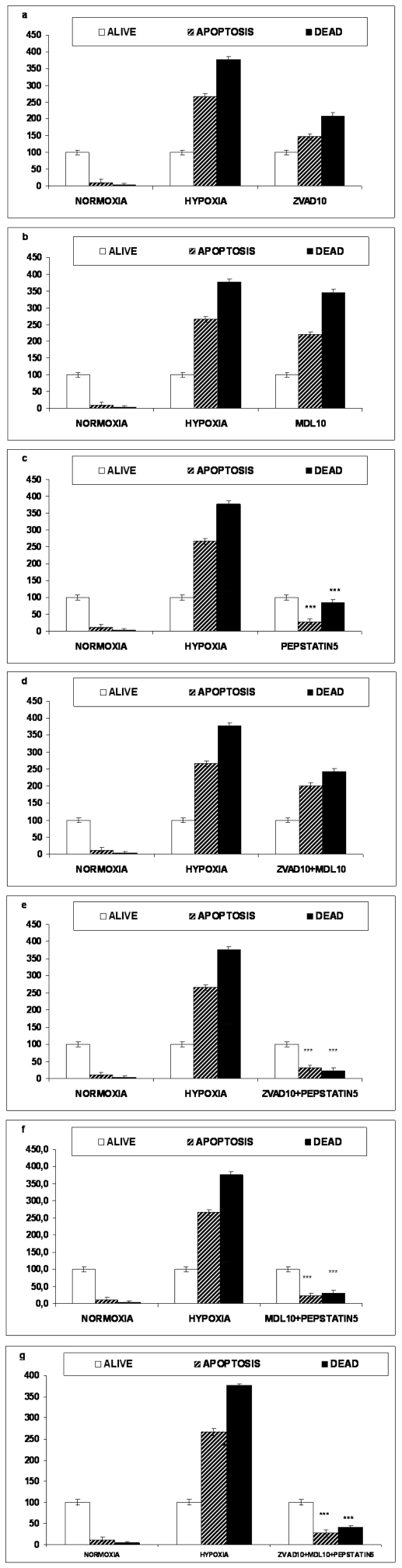
Effect of 16 hours Oxygen-Glucose Deprivation (OGD) on NGF treated PC12 cells, tested with Annexin V–FITC/PI cell membrane permeability assay, after caspase (10 µM), calpain (10 µM), aspartyl protease (5 µM) inhibition and their combinations. Panels a to g. The values are presented as mean ± SEM, *p<0.05, **p<0.01, ***p<0.001.

16 hours OGD exposed NGF treated PC12 cells, were found to be 266.6% apoptotic and 377.1% dead, expressed as percent of the living cell population **Figure 6**. Preincubation with either 10 µM z-VAD-fmk or 10 µM MDL28170 alone or in combination decreased slightly the numbers of both the apoptotic and dead cells but this reduction was not statistically significant **Figures 6 a, b and c**. In sharp contrast, preincubation with 5 µM pepstatin either alone or in combination with the other inhibitors was sufficient to protect NGF treated PC12 cells from lethal hypoxia in a statistical significant manner. 5 µM pepstatin treatment alone reduced the apoptotic and dead cell percentages to 27.8% and 85.3% respectively (p<0.001) **Figure 6d**. 5 µM pepstatin in combination with z-10 µM VAD-fmk treatment reduced the apoptotic and dead cell percentages to 31.9% apoptotic and 23.4%respectively (p<0.001) and 5 µM pepstatin in combination with 10 µM MDL28170 reduced the cell percentages to 21.7% apoptotic and 30.7% dead (p<0.001). When the triple inhibitors combination treatment was employed, the percentages were as follows: 28.1% apoptotic and 41.2% dead (p<0.001) **Figure 6 e, f, and g**.

## Discussion

In this work we investigated the involvement of different classes of proteases in the cell death elicited by OGD on naive and NGF treated PC12 cells. In the past, there were reports implicating NGF in the way PC12 cells respond under hypoxic conditions or that NGF exerts a neuroprotective role in a PC12 in vitro OGD model [29], [30], therefore we felt it would be important to extend our experimentation on NFG treated PC12 cells in our OGD model and test the consistency of our observations in the two different developmental stages of the PC12 cells. We have preincubated the cells with specific protease inhibitors and we subsequently exposed them to OGD in order to assess the involvement of the different protease classes to the OGD induced cell death. Cell death or survival was assessed by the two different assays; the MTT assay measuring the integrity of mitochondrial function and by Annexin V-FITC and PI staining coupled by flow cytometry measuring the integrity of the cell membranes. Trypan blue exclusion assay was used to monitor cell death routinely since there are reports showing that at least in some cases results obtained for cell viability differ significantly depending on the method used [31]. Both trypan blue and MTT assays detected cell death efficiently and the results of both assays were consistent to one another. Our data is in agreement with other reports employing the trypan blue assay for cell viability [32].

Three major classes of proteases are involved in cell death and these are the caspases, the calpains and the lysosomal cathepsins. Out of these classes both caspases and calpains are cysteine proteases while the lysosomal cathepsins are serine, aspartyl, as well as cysteine proteases. We chose to use three different protease inhibitors “specific” to the different types of cell death. z-VAD-fmk, MDL28170 and pepstatin A. Since inhibition of protease activity is of the outmost importance pertaining to the design of this work we took great care to ensure that each protease inhibitor is specific to each class of proteases involved. Information about the specificity of protease inhibitors employed was primarily from the MEROPS Database [33]. z-VAD-fmk is a specific inhibitor of caspases (cysteine proteases central to the apoptotic mechanism). We stress out the fact that at 10 µM concentration used herein, z-VAD-fmk is a caspase-specific inhibitor, while at higher concentrations (50–100 µM) it can also abolish the activity of the cathepsins (cysteine lysosomal proteases B and L) [34]. MDL28170 was used as a specific inhibitor of calpains (calcium dependent cysteine proteases involved in necrotic cell death) that can also inhibit the lysosomal cysteine protease cathepsin B, albeit to a lesser extent and with lower affinity for it (K_i_ values are 10 and 25 nM, respectively), [35], [36]. Lastly, pepstatin A was used as a specific inhibitor for aspartyl proteases including the lysosomal aspartyl proteases cathepsins D and E [34].

Our data show that inhibition of the caspase activity with 10 µM z-VAD-fmk rescues PC12 cells from OGD induced apoptosis (**Figure 1a**). Higher concentrations of the inhibitor were not as effective in protecting the cells. Similarly, inhibition of the calpains by 10 µM or 50 µM MDL28170 rescues PC12 cells from OGD elicited cell death (**Figure 2a**). Similar results were obtained when using the same inhibitors with PC12 cells differentiated with NGF indicating that the cell death or survival mechanism was passed into the differentiated phenotype (**Figures 1b** **and** **2b**). Since we find that both inhibitors rescue the cells from OGD elicited cell death indicates that i) both apoptosis and necrosis are two individual and separate components of the OGD elicited cell death or ii) both apoptosis and necrosis share components of their machinery to one another. In fact the latter hypothesis is supported by the results from the experiments employing combinations of the inhibitors used (figures 4a and 4b). This is also supported by other reports. For example the calpain-cathepsin hypothesis in which calpain mediated lysosomal rupture and release of cathepsin B initiates necrosis [37], [38]. Additionally, other reports suggest that cathepsin B is required for the initiation of apoptosis [23], [24], [39]. When we used pepstatin A as a specific inhibitor of cathepsins D and E, alone or in combination, with the other inhibitors we observed protection from OGD in all cases (**Figures 3a** **,** **4a**). The same results were obtained with the NGF differentiated PC12 cells (**Figures 3b** **and** **4b**).

Using flow cytometry we were not able to detect any protection from OGD on naive or NGF treated PC12 cells applying caspase or calpain inhibitors, alone or in combinations to each other (**Figures 5a,b,d** **and** **6a,b,d**). When pepstatin A was used to protect from proteolysis, alone or in combinations with the other inhibitors, we were able to demonstrate protection from OGD and this was observed both on naive PC12 and NGF treated cells (**Figures 5 c,e,f,g and 6 c,e,f,g**). Inhibition with pepstatin A lowered the percentages of the dead and apoptotic cell population and raised the percentage of the living cells.

We argue that the failure to detect any protection while using apoptosis or necrosis specific inhibitors is relevant to the extent of the mobilisation of these mechanisms. However, since only the inhibition of aspartyl proteases provided significant protection of PC12 exposed to OGD in all cases we propose that the lysosomal cathepsins D and E may have a distinct role in the cell death elicited by OGD.

This role is central both on naive and NGF treated PC12 cells indicating that the mechanism(s) involved are conserved through the NGf induced differentiation process.

The role of the aspartyl proteases and in particular of cathepsin D in the different cell death pathways has been subject for intense investigation. Cathepsin D is reported to promote or to even to inhibit apoptosis in certain cases, while in others, it promotes necrosis [25], [40], [41]. Thus, until now, the role of cathepsin D remains controversial.

Autophagy is currently emerging as a third way of controlled cell death. Autophagy is a highly organised cellular process aiming mainly in the recycling of misfolded or aged proteins or protein aggregates. The pathway evolved in unicellular eukaryotes as means to conserve energy in times of food shortage. There are different subclasses of autophagy such as macroautophagy, microautophagy and chaperon-mediated macroautophagy. In all cases the cellular parts or protein aggregates are driven into the lysosome for degradation. The pathway of autophagy has been implicated in many neurodegenerative diseases [42] and the interplay of autophagy with the established mechanisms of cell death i.e. apoptosis and necrosis is now apparent [27]. There are even recent reports stating that autophagy is involved in ischemic preconditioning [8].

As is evident from the above, approaches to understand the response of PC12 cells to OGD by means of proteolysis inhibition are extremely complex. Not only calpain mediated necrosis leads to lysosome rupture and release of cathepsin B but also apoptosis seems to be regulated by the cathepsin B as well. One the other hand our approach removes the magnifying glass of the scientist and takes a peek to a bigger picture pertaining to the participation of each class of proteases tested to the cell death elicited by OGD. Our data shows that mobilisation of caspases or calpains have limited effect in the death induced by OGD and indicate a distinct role for the lysosomal aspartyl cathepsins D and E. We propose that in view of the recent advances in the field of protein turn over and degradation future research should focus towards understanding the role of aspartyl proteases in OGD elicited cell death.

## Materials and Methods

### Cell cultures

All experiments were performed with rat pheochromocytoma (PC12) cells a generous gift from I. Papamatheakis (Institute of Molecular Biology and Biotechnology, Foundation of Research and Technology Heraklion Crete, Greece). Naive PC12 cells were cultured in collagen-coated dishes with High Glucose Dulbecco's Modified Eagle Medium (DMEM 4.5 g/l glucose) (Gibco) supplemented with 2.5% heat-inactivated foetal bovine serum (Gibco), 15% heat-inactivated horse serum (Gibco) 100 U/ml penicillin and 100 µg/ml streptomycin, in vented culture flasks (BD). The cultures were maintained in 5% CO_2_–95% humidified air at 37°C. Medium was changed every 2–3 days. By 3 to 4 days of incubation in the flask, cells had reached 70–80% confluence and were seeded onto a 96-well flat bottom microtitre plate at a cell density of 1×10^5^/well for testing [43], [44].

### Cell viability

Routine assessment of cell viability was determined using the trypan blue exclusion assay. Living cells (not stained by trypan blue, Sigma) were counted on a Neubauer plate.

### NGF Differentiation

PC12 cells were seeded in 96-well plates (corning) at a density of 10^5^ cells/well, in low glucose medium (DMEM 1000 mg/ml glucose) with 100 ng/ml rat recombinant 2.5S NGF (R&D). Medium and NGF were replenished every 2–3 days. Experiments were performed after 7–8 days of NGF-induced differentiation. All conditions were performed in triplicates. Medium changes were performed at least 24 h before any experiments in NGF-treated cells, to ensure that a basal state of signalling had been reached.

### Lethal Hypoxia-OGD

Lethal hypoxia was induced by exposing PC12 cultures to a calibrated gas mixture of 2%CO_2_, 5% CO_2_, 93% N_2_ in a 3-gas incubator (Forma Scientific), in complete medium (DMEM) depleted of glucose and sodium pyruvate, for 16 hours, according to protocols described by [8], [45]. Control cells were maintained in normoxia (5% CO2–95% humidified air), in complete low glucose medium (DMEM 1,0 g/l).

### MTT assay

Integrity of cellular function was measured by the MTT (3-(4,5- dimethylthiazol-2-yl)-2,5-diphenyltetrazolium bromide; Sigma-Aldrich) tetrazolium salt assay. This colorimetric assay is based on the capacity of the mitochondrial enzyme succinate dehydrogenase to reduce the yellow MTT tetrazolium salt into blue MTT formazan crystals by living cells. The level of conversion provides an indication of mitochondrial metabolic function. MTT, according to the manufacturer's instructions, was dissolved in DMEM without phenol red at a final concentration of 5 mg/ml, and served as a stock solution. PC12 cells exposed to OGD were further incubated for 4 h with 1 mg/ml MTT at 37°C in a normoxic chamber. At the end of the incubation period MTT formazan crystals were solubilised with 0.1 N HCl isopropanol and absorption was detected at 570 nm, with background subtraction at 630 nm, using a micro-plate reader (Stat Fax – 2100, Awareness Technology Inc. USA) [43], [44], [46].

### Flow cytometry

To detect the translocation of phosphatidylserine from the inner face of the cell membrane to the outer surface, we used the Annexin V–FITC cell membrane labelling assay as an early marker of apoptosis. In order to label the DNA in cells where the cell membrane had been compromised we used Propidium iodide (PI). The assay was performed using an Annexin V–FITC Apoptosis Detection Kit according to the manufacturer's instructions (BD).

Cells were collected, washed in PBS and re-suspended in binding buffer and a 0.5 ml aliquot was withdrawn for analysis. After addition of Annexin V–FITC and PI, the sample was incubated for 10 min in the dark. Stained cells were analysed on a BD flow cytometer, excitation was at 488 nm and green and red emission filters were at 515–545 nm and 600 nm respectively. A total of 10,000 cells were counted per sample, and the data were processed using standard software.

Routinely, the MTT and the Annexin V-FITC/PI staining assays for flow cytometry were performed in parallel in twin cultures that were subjected to identical conditions. This procedure was adopted in order to eliminate variations of the cell population, growth conditions and experimental procedures.

### Inhibitors

Caspase activity following oxygen and glucose deprivation was tested using zVAD-fmk (*N*-benzyloxycarbonyl-Val-Ala-Asp-fluoromethylketone - Sigma), a cell permeable, irreversible caspase inhibitor. zVAD-fmk was prepared as 500×stocks in dimethylsulfoxide and applied at 10 µM and 50 µM concentrations to PC12 and NGF treated cells 30 min before oxygen and glucose deprivation treatment.

We tested also the effects of a calpain inhibitor MDL28170 (Cbz-Val-Phe-CHO - Sigma) after oxygen and glucose deprivation. The compound, MDL28170, is a membrane-permeable cysteine protease inhibitor which potently inhibits both calpain I and II. MDL28170 was made as 500×stock solution in dimethylsulfoxide and applied at 10 µM, 50 µM and 100 µM concentrations 30 min before OGD treatment.

Aspartyl-protease involvement in cell damage by OGD was investigated using pepstatin A, a lysosomal aspartic protease inhibitor. Pepstatin A (Sigma) was prepared as 1 mg/ml in 10% (v/v) acetic acid in methanol (9∶1 methanol∶ acetic acid), and applied at 1 µM and 5 µM concentrations to PC12 and NGF treated cells 30 min before oxygen and glucose deprivation treatment.

### Statistical analysis

The computer software SPSS 16.0 (SPSS Inc.) was used for all statistical calculations and analyses. All data were analyzed with analysis of variance (ANOVA) for repeated measurements. If significant, ANOVA was followed by post hoc multiple comparisons with CTRL and ND group by Dunett's test. Two-tailed levels of significance were used in all statistical calculations. Statistical significance level for group differences was set at P≤0.05. All data are expressed as mean ± standard error of the mean (SEM) of separate experiments *n*≥3. Significant differences were indicated by *p = 0.05, **p = 0.01 and ***p = 0.001 with respect to control or a corresponding single treatment.
